# Production of 3-hydroxypropionic acid from glucose and xylose by metabolically engineered *Saccharomyces cerevisiae*

**DOI:** 10.1016/j.meteno.2015.10.001

**Published:** 2015-10-31

**Authors:** Kanchana R. Kildegaard, Zheng Wang, Yun Chen, Jens Nielsen, Irina Borodina

**Affiliations:** a The Novo Nordisk Foundation Center for Biosustainability, Technical University of Denmark, Kogle Allé 6, 2970 Hørsholm, Denmark; b Department of Biology and Biological Engineering, Chalmers University of Technology, Göteborg, Sweden; c The Novo Nordisk Foundation Center for Biosustainability, Chalmers University of Technology, Göteborg, Sweden

**Keywords:** 3HP, 3-hydroxypropionic acid, XR, xylose reductase, XDH, xylitol dehydrogenase, XK, xylulokinase, MCR, malonyl-CoA reductase, ACC, acetyl-CoA reductase, ALD, aldehyde dehydrogenase, ACS, acetyl-CoA synthase, PDC, pyruvate decarboxylase, PYC, pyruvate carboxylase, BAPAT, β-alanine-pyruvate aminotransferase, PAND, aspartate 1-decarboxylase, HIBADH, 3-hydroxyisobutyrate dehydrogenase, HPDH, 3-hydroxypropionate dehydrogenase, Metabolic engineering, Biorefineries, 3-hydroxypropionic acid, *Saccharomyces cerevisiae*, Xylose utilization

## Abstract

Biomass, the most abundant carbon source on the planet, may in the future become the primary feedstock for production of fuels and chemicals, replacing fossil feedstocks. This will, however, require development of cell factories that can convert both C6 and C5 sugars present in lignocellulosic biomass into the products of interest. We engineered *Saccharomyces cerevisiae* for production of 3-hydroxypropionic acid (3HP), a potential building block for acrylates, from glucose and xylose. We introduced the 3HP biosynthetic pathways via malonyl-CoA or β-alanine intermediates into a xylose-consuming yeast. Using controlled fed-batch cultivation, we obtained 7.37±0.17 g 3HP L^−1^ in 120 hours with an overall yield of 29±1% Cmol 3HP Cmol^−1^ xylose. This study is the first demonstration of the potential of using *S. cerevisiae* for production of 3HP from the biomass sugar xylose.

## Highlights

• 3HP is a potential building block for acrylic plastics and biodegradable polyesters.
• We developed *S. cerevisiae* strains for production of 3HP from glucose and xylose.
• 3HP pathway via β-alanine resulted in the highest 3HP titers on xylose.
• 7.4±0.2 g 3HP L^−1^ was obtained with a yield of 29±1% Cmol 3HP Cmol^−1^ xylose.
• Demonstration of the potential of using the biomass sugar xylose to produce 3HP.

## 1 Introduction

In order to maintain and improve our current living standard it is necessary with a transition to a sustainable fossil-free society, and this will require novel technologies for production of energy, fuels and chemicals. In the absence of strong governmental and regulatory support, the emerging bio-based technologies must compete with the established petrochemical industry. Nevertheless during the last decade we have witnessed positive developments in the bio-based industry: appearance of the first commercial-scale 2nd generation ethanol biorefineries and of commercial plants for bulk bio-based chemicals, such as lactic acid, farnesene, isobutanol, 1,4-butadiene, etc. (see Borodina and Nielsen (2014), Li and Borodina (2014) and Van Dien (2013) for references and details). With increasing bio-based production it will not be possible to solely use sugars from food crops as a feedstock, and there is therefore a need for creating novel cell factories that can produce chemicals from lignocellulosic materials, which contain both pentoses (C5) and hexoses (C6).

3-Hydroxypropionic acid (3HP) is an attractive bio-based platform chemical as it can be chemically converted into acrylic acid, acrylic esters and amides, and hereby enables sustainable production of superabsorbent polymers, plastics, paints, etc. The market for acrylic acid and its esters is estimated to reach USD 18.8 billion by 2020 (“Acrylic Acid Market (Acrylate Esters, Glacial Acrylic Acid & Others) for Superabsorbent polymers and Surface Coatings, Adhesives and Sealants, Textiles, Plastic Additives and Printing Ink Applications-Global Industry Analysis, Size, Share, Growth and Forecast, 2012–2018,”). 3HP has been produced biologically from glycerol by *Klebsiella pneumoniae* and *Escherichia coli* strains, from glucose at neutral pH by *E. coli* (see Kumar et al. (2013) for references and details), and lately also from glucose at lower pH by yeast *Saccharomyces cerevisiae* (Borodina et al., 2015, Chen et al., 2014, Jensen et al., 2014a). As 3HP is an organic acid with pKa of 4.51, running the fermentation at low pH reduces the downstream process cost and amount of waste, and a yeast-based process is therefore preferable. Furthermore, yeast has an advantage of being tolerant to inhibitors in biomass hydrolysate and can be engineered for tolerant to high concentration of 3HP (Kildegaard et al., 2014). Here we explored the production of 3HP from d-xylose by engineered *S. cerevisiae*.

## 2 Materials and methods

### 2.1 Strains and chemicals

The xylose utilizing *S. cerevisiae* strain was described before (Scalcinati et al., 2012). Recombinant yeast strains were selected and maintained on synthetic drop-out agar without tryptophan and with 2% xylose as the sole carbon source. Chemicals were purchased from Sigma-Aldrich. 3HP was purchased from Tokyo Chemical Industry Co. (TCI). Pfu Turbo DNA polymerase was from Agilent Technologies Inc.

### 2.2 Strain construction

The yeast strains and plasmids are listed in Table 1. The primers and biobricks are listed in Appendix A, Appendix A, respectively. The xylose-consuming CMB.GS010 strain was made auxotrophic for uracil, histidine and leucine as following. First, the ORF of *URA3* gene was replaced with the *KanMX* cassette using homologous recombination. As the *KanMX* cassette is flanked by LoxP sites, the *KanMX* cassette was removed by Cre-LoxP-mediated selection marker loop-out as described previously (Jensen et al., 2014b). Next, the ORF of *LEU2* and *HIS3* genes were replaced by the *URA3* from *Kluyveromyces lactis* (*KlURA3*) and *KanMX* cassettes, respectively. Finally, the *KlURA3* and *KanMX* cassettes were looped-out using Cre-LoxP system to generate the final strain ST2488. The yeast transformations were performed using the lithium acetate protocol (Gietz and Schiestl, 2007). The elimination of the selection markers was verified by PCR in addition to phenotypic test. To construct 3HP-producing strains, expression vectors carrying the genes involved in 3HP biosynthesis were linearized with NotI and then transformed into ST2488. The transformants were selected on synthetic drop-out xylose medium without uracil, histidine, leucine and tryptophan.

**Table 1:** Strains and plasmids used in this study.

**Plasmids**	**Description**	**Reference**
pUG6	LoxP-KanMX-LoxP	Euroscarf
pUG72	LoxP-KlURA3-LoxP	Euroscarf
pCfB380	pX-3-LoxP-KlLEU2-SEacs^L641P^<-P_TEF1_-P_PGK1_->ALD6	Jensen et al. (2014a)
pCfB382	pX-4-LoxP-SpHiS5-PDC1<-P_TEF1_	Jensen et al. (2014a)
pCfB474	pTY4-KlURA3-ACC1^**^<-P_TEF1_-P_PGK1_->CaMCR	Jensen et al. (2014a)
pCfB743	pXI-1-LoxP-KlLEU2-PYC1<-P_TEF1_-P_PGK1_->PYC2	Borodina et al. (2015)
pCfB799	pTY4-TcPanD<-P_TEF1_	Borodina et al. (2015)
pCfB800	pX-4-LoxP-SpHiS5-BcBAPAT<-P_TEF1_-P_PGK1_->EcYdfG	Borodina et al. (2015)
pCfB801	pX-4-LoxP-SpHiS5-BcBAPAT<-P_TEF1_-P_PGK1_->PpHIBADH	Borodina et al. (2015)
		
**Strains**	**Genotype**	**Reference**
CEN.PK 113-7D (ST1)	*MATa URA3 HIS3 LEU2 TRP1 MAL2-8*^*c*^ *SUC2*	Peter Kötter (Johann Wolfgang Goethe-University Frankfurt, Germany
CMB.GS010	CEN.PK 113-3C/pRS314-X123 (P_TDH3_-*PsXYL1*, P_TDH3_-*PsXYL2*, P_TDH3_-*PsXYL3 TRP1*) evolved for grown on xylose	Scalcinati et al. (2012)
ST2488	CMB.GS010 *TRP1 ura3∆ his3∆ leu2∆*	This study
ST2546	ST2488/pCfB380/ pCfB382/ pCfB474	This study
ST2547	ST2488/pCfB743/ pCfB800/ pCfB799	This study
ST2808	ST2488/pCfB743/ pCfB801/ pCfB799	This study

### 2.3 Cultivation of yeast

For testing 3HP production, the strains were cultivated in mineral medium with 20 g L^−1^ glucose or xylose as carbon-source in 96-deep well plate as described earlier (Borodina et al., 2015).

Controlled fermentations were carried out according to the following protocol. Batch fermentations were performed in the mineral media as previously described (Verduyn et al., 1992) containing 50 g L^−1^ of xylose, 5 g L^−1^ of (NH_4_)_2_SO_4_, 3 g L^−1^ of KH_2_PO_4_, 0.5 g L^−1^ of MgSO_4_·7H_2_O, 0.05 mL of antifoam, 1 mL of a vitamin solution and 1 mL of a trace metal solution. The medium used to prepare the pre-cultures in shake-flasks was the same as above with the following modifications: no antifoam, 7.5 g L^−1^ of (NH_4_)_2_SO_4_, 14.4 g L^−1^ of KH_2_PO_4_ and the pH was adjusted to 6.5 with NaOH before autoclaving. Each fermenter was inoculated with an initial OD_600_ of 0.5 using a pre-culture obtained by cultivating a single colony of the desired strain in 30 mL of mineral medium in a 100 mL shake-flask at 200 rpm in an orbital shaker kept at 30 °C. The batch and fed-batch fermentations were performed in 2.7-L DASGIP Bioreactors (Dasgip, Jülich, Germany). The working volume for batch fermentations was 1 L, the temperature set-point was controlled at 30 °C, the airflow was set at 1 vvm (gas volume flow per unit of liquid volume per minute), the pH was maintained at 5 by feedback controlled addition of 10% NH_4_OH, the dissolved oxygen was kept above 30% of saturation by feedback control of the stirring speed from 600 rpm until a maximum of 1200 rpm. The concentration of O_2_ and CO_2_ in exhaust gas was monitored by a DASGIP® GA4 exhaust analyzer. The fed-batch cultures were initiated as batch cultures using 20 g L^−1^ xylose. An exponential feeding rate of xylose was designed to keep the growth rate at 0.03 h^−1^.

### 2.4 Analysis of biomass and metabolites

The growth was measured by optical density at 600 nm and cell dry weight. Extracellular metabolites, such as 3HP, glucose, xylose, ethanol, acetate, glycerol, and succinate, were analyzed by HPLC as reported in (Borodina et al., 2015).

## 3 Results and discussion

### 3.1 Engineering 3HP pathways into the xylose-consuming yeast

The xylose consuming yeast CMB.GS010 was constructed previously by transforming CEN.PK 113–3C with the centromeric vector pRS314-X123 carrying xylose reductase (XR), xylitol dehydrogenase (XDH) and xylulokinase (XK) from *Pichia stipitis* under control of strong constitutive promoter *TDH3* and evolving the resulting strain on mineral medium with xylose as the sole carbon source (Scalcinati et al., 2012). The evolved strain had an increased maximal specific growth rate compared with the non-evolved parent strain, i.e. 0.18 h^−1^ compared with 0.02 h^−1^, and showed a 15-fold increase in the xylose consumption rate. As the CMB.GS010 strain has no selectable markers available for further genetic modifications, three auxotrophic mutations (*ura3Δ*, *leu2Δ and his3Δ*) were made in strain CMB.GS010 to obtain strain ST2488.

**Fig. 1:**
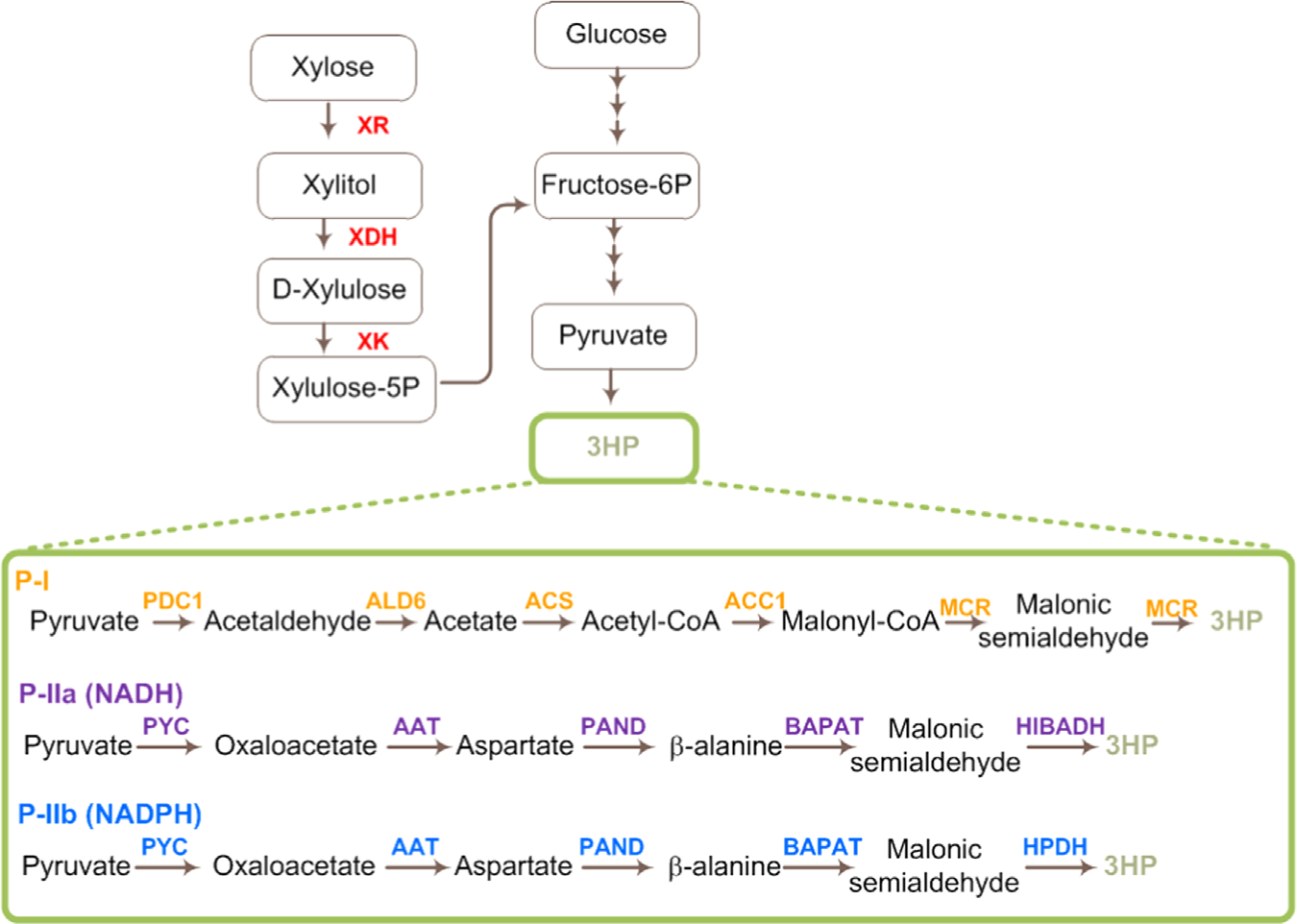
Overview of the pathway for xylose utilization and 3HP biosynthesis. P-I denotes a pathway towards 3HP via malonyl-CoA intermediate, P-II is a pathway towards 3HP via β-alanine intermediate.

**Fig. 2:**
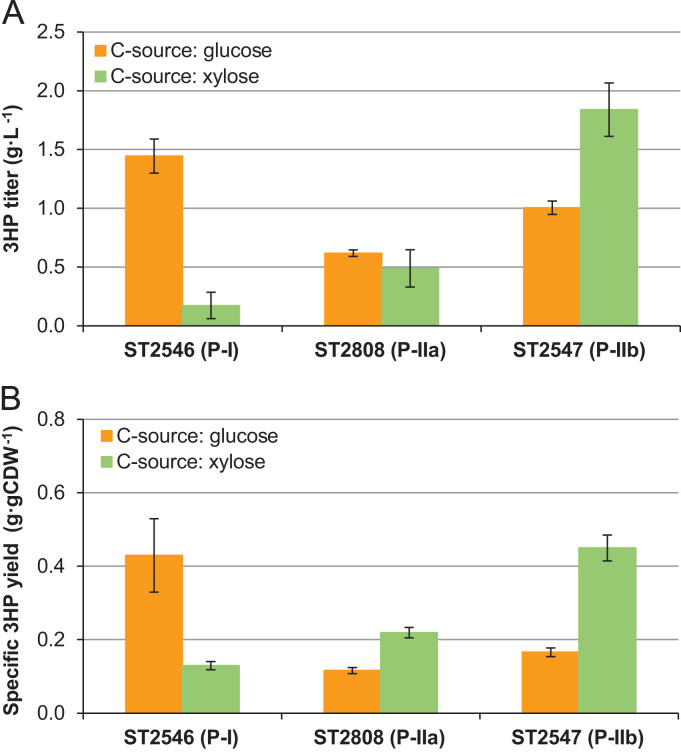
3HP production from glucose or xylose by the engineered *S. cerevisiae* strains, carrying biosynthetic pathways towards 3HP via malonyl-CoA or β-alanine intermediates. (A) 3HP titers and (B) 3HP yields from 15 individual transformants that were cultivated on both substrates for each strain. The average values and standard deviations are shown.

In ST2488, we introduced two different pathways for 3HP production, one via malonyl-CoA (P-I) and another via β-alanine (P-II) (Jensen et al., 2014a; Borodina et al., 2015) (Fig. 1). The strain with P-I (ST2546) carried overexpression cassettes for 5 genes: malonyl-CoA reductase from *Chloroflexus aurantiacus MCR*, phosphorylation insensitive variant of acetyl-CoA carboxylase *ACC1*^*S659A, S1157A*^ (ACC1^**^), aldehyde dehydrogenase (*ALD6*), acetyl-CoA synthase from *Salmonella enterica* (*SE*_*ACS*_), and pyruvate decarboxylase (*PDC1*). *MCR* and ACC1^**^ were integrated into TY4 retrotransposon regions using degradation-tagged URA3 selection marker as described in (Borodina et al., 2015) to ensure multiple integration events. The pathway P-II either used a NADH-dependent route (P-IIa) or a NADPH-dependent route (P-IIb). The strain with P-IIa (ST2808) carried over-expression cassettes for 5 genes: pyruvate carboxylase (*PYC1*, *PYC2*), β-alanine-pyruvate aminotransferase from *Bacillus cereus* (*BcBAPAT*), aspartate 1-decarboxylase from *Triboleum castanium* (*TcPAND*), and NADH-dependent 3-hydroxyisobutyrate dehydrogenase (HIBADH) from *Pseudomonas putida* (*PpHIBADH*). In the strain with P-IIb (ST2547) the last gene was replaced by NADPH-dependent 3-hydroxypropionate dehydrogenase (HPDH) from *E. coli* (*EcYdfG*). In the strains with P-IIa and P-IIb pathways, all the genes were integrated into the genome in a single copy, with exception of *TcPAND* gene, which was inserted into TY4 regions. Because the resulting transformants may have different copy numbers of the vectors integrating into different TY4 regions, we chose to screen 15 transformants of each strain for 3HP production on glucose and xylose. The average titer on glucose was highest for the strain with P-I (1.44±0.14 g L^−1^), however this strain had a very poor 3HP production on xylose (0.17±0.11 g L^−1^) (Fig. 2). The 3HP titers for the P-IIa strain were comparable on glucose and xylose (0.60±0.03 and 0.49±0.16 g L^−1^) and the specific yield on xylose was nearly twice as high as that on glucose. The best titer was obtained for strain with P-IIb pathway, 1.00±0.06 g L^−1^ on glucose and 1.84±0.23 g L^−1^ on xylose and the specific yield on xylose was nearly 2.7-fold higher than that on glucose. We speculate that the reason for low 3HP production from xylose via P-I is the lack of overflow metabolism (Crabtree effect), where a large part of the carbon is channeled via pyruvate decarboxylase into ethanol. As was shown in a previous study (Borodina et al., 2015), 3HP accumulation largely occurs during the ethanol consumption phase during cultivation on glucose, however only very limited amounts of ethanol are produced during growth on xylose. The absence of the Crabtree effect had however a positive effect on the 3HP production via the β-alanine pathway, which begins with the anaplerotic reaction from pyruvate to oxaloacetate, and hence competes for pyruvate with pyruvate decarboxylase.

### 3.2 Production of 3HP in controlled batch and fed-batch reactors

**Fig. 3:**
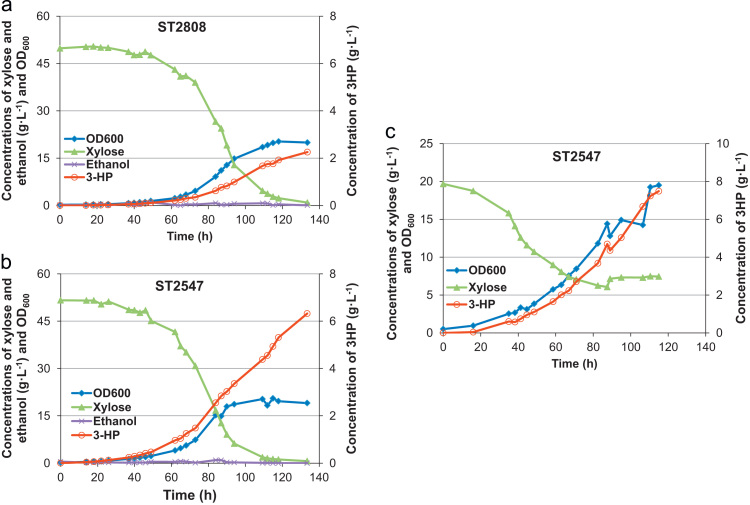
Physiological characterization of 3HP-producing strains. (A) Batch fermentation of strain ST2808. (B) Batch fermentation of strain ST2547. (C) Fed-batch fermentation of strain ST2547. The strains were grown on mineral medium with xylose as the sole carbon source at pH 5. Each fermentation was carried out in duplicates. Here a representative graph is shown (the replica data is provided in Supplementary Fig. 1).

The best 3HP-producing strains ST2547 and ST2808 were fermented in batch mode on mineral medium with xylose as the sole carbon source (Fig. 3). The pH was maintained at 5 during the fermentation. In batch mode about 50 g L^−1^ of xylose was consumed in 140 hours, resulting in 6.09±0.33 g L^−1^ 3HP by ST2547, compared with 2.3±0.09 g L^−1^ 3HP by ST2808. Ethanol concentration did not exceed 1 g L^−1^ at any point of fermentation. ST2547 strain was further characterized in fed-batch mode. In fed-batch mode after 120 hours of fermentation, the 3HP concentration reached 7.37±0.17 g L^−1^, with an overall yield of 29±1% C-mol 3HP C-mol^−1^ xylose. As xylose does not elicit the Crabtree effect, the batch or fed-batch mode of sugar addition did not have a significant impact on the final titer or yield.

## 4 Conclusions

3HP production via two different pathways has been established in the evolved xylose-utilizing yeast, where the 3HP pathway via β-alanine resulted in higher product titer on xylose than the pathway via malonyl-CoA. The study lays the basis for development of the yeast strain for producing 3HP from lignocellulosic feedstocks at low pH.

## Footnotes

Appendix A Supplementary data associated with this article can be found in the online version at doi:10.1016/j.meteno.2015.10.001.
