# Supplementary material for: Production of 3-hydroxypropionic acid from glucose and xylose by metabolically engineered Saccharomyces cerevisiae
Source: Metab Eng Commun. 2015 Oct 31;2:132–6. doi: 10.1016/j.meteno.2015.10.001 (PMC8193239; doi:10.1016/j.meteno.2015.10.001)
Supplement: Supplementary file 1 — Supplementary material [file mmc1.docx]

**Appendix A. Supplementary materials**

**Supplementary Figure**

**Supplementary Fig. 1.**  Batch and fed-batch fermentations of 3HP-producing strains. The cultivations were performed in duplicates. Here the replicates of fermentations from Figure 3 are shown. (A) Batch fermentation of strain ST2808. (B) Batch fermentation of strain ST2547. (C) Fed-batch fermentation of strain ST2547. All the strains were grown on mineral medium with xylose as the sole carbon source at pH 5.

**Supplementary Tables**

**Supplementary Table 1.** Primers used in this study

| **Primer name** | **Sequence (5’ ->3’)** |
| --- | --- |
| **Knock-out primers** | |
| ID91_URA3_2/3_START_fw | TGGCAATTCCCGGGGATC |
| ID92_URA3_2/3_START_rv | CGCTTCCCATCCAGCATTTC |
| ID93_URA3_2/3_END_fw | CTGTCGTTCCATTGAAAGC |
| ID94_URA_2/3_END_rv | TAGGGCGAATTGGGTACC |
| ID476_KanMX_2/3_START_fw | TGGCAATTCCCGGGGATCACGCTGCAGGTCGACAAC |
| ID477_KanMX_2/3_START_rv | AGTGACGACTGAATCCGGTG |
| ID478_KanMX_2/3_END_fw | AATGGGCTCGCGATAATGTC |
| ID479_KanMX_2/3_END_rv | TAGGGCGAATTGGGTACCGCCACTAGTGGATCTGATATCA |
| ID480_LEU2_UP_fw | ACATCGAGACCAAGAAGAAC |
| ID481_LEU2_UP_rv | GATCCCCGGGAATTGCCAGCTGAAATGTAAAAGGTAAGAAAAG |
| ID482_LEU2_DOWN_fw | GGTACCCAATTCGCCCTAGAAGAAGTTAAGAAAATCCTTGC |
| ID483_LEU2_DOWN_rv | GATTTACTATCAATTTCTTCTCACAAG |
| ID484_URA3_UP_fw | GGAGAATCCATACAAGAAATCG |
| ID485_URA3_UP_rv | GATCCCCGGGAATTGCCAGATTTATCTTCGTTTCCTGC |
| ID486_URA3_DOWN_fw | GGTACCCAATTCGCCCTAGTAAATGCATGTATACTAAACTC |
| ID487_URA3_DOWN_rv | GTTATCAGATATTATCAGGTGG |
| ID698_HIS3_UP_fw | GAATAGTGCCATGGTACAGC |
| ID699_HIS3_UP_rv | GATCCCCGGGAATTGCCAATGTGATAATGCCAATCGC |
| ID700_HIS3_DOWN-fw | GGTACCCAATTCGCCCTATGACACCGATTATTTAAAGCTG |
| ID701_HIS3_DOWN_rv | TGGAGGTGAGGTAAATTCT |
| ID142_marker-DW-rev | AGGCCACUAGTGGATCTGATATCAC |
| ID151_KlLEU2-DW-fwd | TGGAAGAGGCAAGCACGTTAGC |
| **Genotyping primers** |  |
| ID529_LEU2-KO-test-fw | ACAAATGAAGAGAAATTTAGAAAC |
| ID530_LEU2-KO-test-fw | GTCATTCAACCTATTAAAGAAATTG |
| ID531_URA3-KO-test-fw | AGAACTTCATGTGGGTCCTAGG |
| ID532_URA3-KO-test-fw | AACTCTGGGAGCTGCGATTG |
| ID702_HIS3-KO-test-fw | GCTCTGTCGTAACCTTCAGTTC |
| ID703_HIS3-KO-test-rev | CAACGATAGGGACGGAGTAT |
| ID903_X-3-up-out | TGACGAATCGTTAGGCACAG |
| ID905_X-4-up-out | CTCACAAAGGGACGAATCCT |
| ID907_XI-1-up-out | CTTAATGGGTAGTGCTTGACACG |
| ID2221_ColoPCR_vec_TADH1_ towards out | GTTGACACTTCTAAATAAGCGAATTTC |

**Supplementary Table 2.** Primers and templates used to generate gene fragments for USER cloning and yeast transformation by PCR

| **Fragment ID** | **Description** | **Forward primer** | **Reverse primer** | **Template** |
| --- | --- | --- | --- | --- |
| BB94_KanMX_2/3_START | Upper part of LoxP-KanMX-LoxP selection marker cassette | ID476 | ID477 | pUG6 |
| BB95_KanMX_2/3_END | Down part of LoxP-KanMX-LoxP selection marker cassette | ID478 | ID479 | pUG6 |
| BB129_URA3_2/3_START | Upper part of LoxP-KlURA3-LoxP selection marker cassette | ID92 | ID479 | pUG72 |
| BB130_URA3_2/3_END | Upper part of LoxP-KlURA3-LoxP selection marker cassette | ID93 | ID476 | pUG72 |
| BB96_LEU2_UP | Upstream fragment flanking LEU2 gene | ID480 | ID481 | gDNA of  *S. cerevisiae* |
| BB97_LEU2_DOWN | Downstream fragment flanking LEU2 gene | ID482 | ID483 | gDNA of  *S. cerevisiae* |
| BB100_URA3_UP | Upstream fragment flanking URA3 gene | ID484 | ID485 | gDNA of  *S. cerevisiae* |
| BB103_URA3_DOWN | Downstream fragment flanking ura3 gene | ID486 | ID487 | gDNA of  *S. cerevisiae* |
| BB124_HIS3_UP | Upstream fragment flanking HIS3 gene | ID688 | ID689 | gDNA of   *S. cerevisiae* |
| BB125_HIS3_DOWN | Downstream fragment flanking HIS3 gene | ID700 | ID701 | gDNA of  *S. cerevisiae* |
| BB131_LEU2_UP_URA3_2/3_START | Fusion of LEU2_UP and 2/3_URA3_START fragments | ID480 | ID93 | BB96 and BB130 |
| BB132_URA3_2/3_END_ LEU2_DOWN | Fusion of 2/3_URA3_END and LEU2_DOWN fragments | ID92 | ID483 | BB129 and BB97 |
| BB104_URA3_UP_KanMX_ 2/3_START | Fusion of URA3_UP and 2/3_KanMX_START fragments | ID484 | ID477 | BB100 and BB94 |
| BB105_KanMX_2/3_END_ URA3_DOWN | Fusion of 2/3_KanMX_END and URA3_DOWN fragments | ID478 | ID487 | BB95 and BB103 |
| BB127_HIS3_UP_KanMX_ 2/3_START | Fusion of HIS3_UP and 2/3_KanMX_START fragments | ID698 | ID477 | BB124 and BB94 |
| BB128_KanMX_2/3_END_ HIS3_DOWN | Fusion of 2/3_KanMX_END and HIS3_DOWN fragments | ID478 | ID701 | BB95 and BB125 |
